# Airway invasion in non-neurologically ill patients with dysphagia: Contributing factors and associated problems during swallowing process. A retrospective observational study

**DOI:** 10.1097/MD.0000000000022977

**Published:** 2020-11-06

**Authors:** Kang Lip Kim, Gi-Young Park, Dong Rak Kwon, Do Yun Kwon, Sang Gyu Kwak, Hee Kyung Cho

**Affiliations:** a Department of Physical Medicine and Rehabilitation; b Department of Medical Statistics, Catholic University of Daegu School of Medicine, Republic of Korea.

**Keywords:** airway invasion, non-neurogenically, risk factor, videofluoroscopic dysphagia scale, videofluoroscopic swallowing study

## Abstract

Dysphagia can occur among patients receiving medical care despite having no history of neurologic disease. The current study aimed to investigate factors contributing to airway invasion among non-neurologically ill patients with dysphagia.

This retrospective study included 52 non-neurologically ill patients who complained of swallowing difficulty and consulted the Department of Rehabilitation Medicine for videofluoroscopic swallowing studies between January 2018 and June 2019. Patients were then divided into 2 groups according to the presence of airway invasion (penetration or aspiration) based on videofluoroscopic swallowing study findings, with group 1 (n = 26) consisting of patients with airway invasion and group 2 (n = 26) consisting of those without airway invasion. Demographic information, functional ambulation ability within the past 3 months, presence of community acquired pneumonia (CAP), nutritional status, degree of dehydration, history of intensive care unit stay, history of endotracheal intubation, and videofluoroscopic dysphagia scale were reviewed.

Patients with airway invasion exhibited decreased functional ambulation ability, greater incidence of CAP, and lower serum albumin concentration than patients without airway invasion (*P* < .05). Airway invasion among non-neurologically ill patients was significantly associated with functional ambulation ability [odds ratio (OR), 3.57; 95% confidence interval (CI), 1.14–11.19; *P* = .03], serum albumin concentration under 3.5 g/dL (OR, 4.90; 95% CI, 1.39–17.32; *P* = .01), and presence of CAP (OR, 5.06; 95% CI, 1.56–16.44; *P* = .01). Groups 1 and 2 had a videofluoroscopic dysphagia scale score of 37.18 and 16.17, respectively (*P* < .05). Moreover, bolus formation, tongue-to-palate contact, premature bolus loss, vallecular residue, coating of pharyngeal wall, and aspiration score differed significantly between both groups (*P* < .05).

Airway invasion among non-neurologically ill patients was related to decreased functional ambulation ability, lower serum albumin concentration, and presence of CAP. The results presented herein can help guide clinical management aimed at preventing airway invasion among non-neurologically ill patients.

## 1 Introduction

Dysphagia, which is characterized by difficulty in swallowing resulting from oropharyngeal or esophageal conditions, has been a frequent problem following neuromuscular diseases, such as stroke, myasthenia gravis, and Parkinson disease. Structural abnormalities of the oropharynx and esophagus, such as head and neck tumors, Zenker diverticulum, esophageal web, and esophageal ring, have also been shown to cause causes dysphagia.^[1]^ However, some patients who have no neuromuscular diseases can also develop dysphagia. Reports have shown that dysphagia can be caused by decreased physical condition, aging, and neuromuscular diseases. Furthermore, sarcopenia, defined as a decreased in muscle mass and function, has been reported as a risk factor for dysphagia among elderly patients.^[2]^ Nowadays, population aging has been considered a major factor for chronic diseases, leading to increased medical care visits. As such, some of the patients receiving medical care at the hospital might suffer from dysphagia.^[3]^

Given that dysphagia is a considerable clinical condition that can cause life-threatening diseases, accurate evaluation of swallowing function is imperative. Diverse examinations have been developed to assess the degree of swallowing disorders. Though certain clinical bedside tests are accepted,^[4]^ videofluoroscopic swallowing study (VFSS) has been commonly used as the standard test for assessing swallowing dysfunction.^[5,6]^ VFSS can assess the presence of penetration and aspiration (airway invasion) in addition to various problems during the swallowing process, including the oropharyngeal and esophageal phases. Determining the presence of airway invasion via VFSS is important for establishing treatment direction among patients with dysphagia. VFSS results can be converted into objective scores using the videofluoroscopic dysphagia scale (VDS), a 100-point scale initially developed to measure the severity of dysphagia among patients who suffered a stroke.^[5]^ The VDS has been widely used as a quantifiable and reliable indicator of persistent dysphagia after stroke, with studies reporting a specificity and sensitivity of 0.92 and 0.91, respectively.^[5]^ Moreover, the VDS can be used to quantify dysphagia severity in diseases other than stroke.^[7]^

Among the various signs of dysphagia, airway invasion (i.e., the entry of material into the airway) is a significant clinical problem given its potential to cause life-threatening conditions, such as aspiration pneumonia, and subsequently long-term hospitalization.^[3]^ As such, the presence of airway invasion is an important parameter in determining the need for oral feeding (or continuation of tube feeding) among patients with dysphagia. Previous studies have reported that a bedridden state and dehydration,^[8]^ as well as other iatrogenic conditions (hypnotic medications, enteral nutrition, and tracheotomy), were risk factors for aspiration. History of intensive care unit (ICU) stay can also cause swallowing dysfunction and aspiration events.^[9]^ Kim et al reported that the duration of endotracheal intubation was related to aspiration pneumonia and dysphagia after extubation among non-neurologic critically ill patients.^[10]^ Although several studies have reported on risk factors of airway invasion in non-neurologic conditions, few have investigated accompanying problems throughout the swallowing process among patients with dysphagia who have airway invasion.

The current study aimed to determine factors associated with airway invasion according to VFSS findings and identify accompanying problems throughout the swallowing process among non-neurologically ill patients with dysphagia.

## 2 Patients and methods

### 2.1 Patients’ characteristics

**Figure 1:**
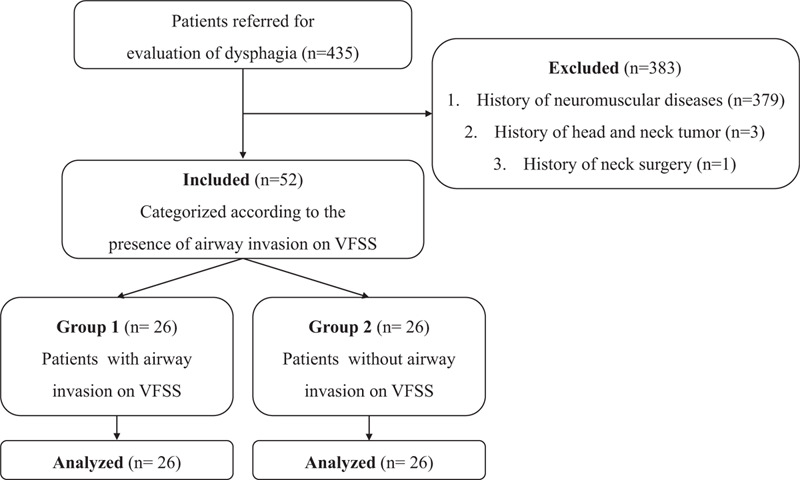
Flow diagram of the study. VFSS = Videofluoroscopic swallowing study.

A retrospective medical chart review was conducted in 435 patients referred to the Department of Physical Medicine and Rehabilitation of the University Medical Center, Daegu, Republic of Korea for evaluation of dysphagia from January 2018 to June 2019. The inclusion criteria were patients aged 18 years and older who underwent VFSS and complied with instructions for performing the same. The exclusion criteria were as follows: a previous history of neuromuscular diseases (e.g., stroke, myasthenia gravis, and Parkinson disease), structural abnormalities of the oropharynx and esophagus (e.g., head and neck tumor, Zenker diverticulum, esophageal web, and esophageal ring), history of neck surgery, inability or difficulty related to VFSS participation, or those at high risk for radiation exposure (e.g., pregnancy). A total of 52 subjects (34 males and 18 females; mean age, 74.4 ± 10.3 years) who satisfied the inclusion and exclusion criteria were included. The study protocol was approved by the Institutional Review Board of Daegu Catholic University Medical Center, in accordance with the Declaration of Helsinki. Given the retrospective nature of the study, informed consent was not required. Patients were then divided into 2 groups according to the presence of penetration and aspiration (airway invasion) based on VFSS findings. (Fig. 1)^[11]^

### 2.2 VFSS analysis

All patients underwent routine VFSS for clinical evaluation by an experienced rehabilitation medicine specialist and a radiologist. VFSS was conducted in the lateral view with continuous fluoroscopy (1 pulse per 90 ms) and recorded at 15 frames per second using SONIALVISION G4. Each patient performed a total of 7 swallows consisting of 3, 6, and 9 mL for both thin and thick liquids via a syringe (10 mL) and a spoon of solid food. Tomato juice (My Love Tomato; WoongJin, Seoul, Korea) and curd-type yogurt (BIO plain sweet; Maeil, Seoul, Korea) were used for the thin and thick liquids, respectively. To make the thin and thick liquid radiopaque, 30 cm^3^ of liquid was mixed with 140% barium sulfate liquid (Solotop Suspension 140; Taejoon Pharm, Seoul, Korea). A spoon of boiled potatoes (approximately 25 g) coated with a thin layer of 140% barium sulfate liquid was provided as solid food. The thick liquid was provided initially, followed by the thin liquid and solid food.^[12]^ Aspiration was defined as passage of materials below the vocal folds, while penetration was defined as passage of materials into the larynx but not below the vocal folds. For safety purposes, the study was immediately ended if massive aspiration developed.^[13]^

VFSS findings were assessed by 2 rehabilitation specialists using a 100-point VDS. The VDS consists of oral and pharyngeal items, each having 7 items, for a total of 14 items. The 14 items are weighted values with a total of 100 points, with a higher score representing worse function.^[5,7]^ The highest VDS score in each case was selected and evaluated.

### 2.3 Data collection

Data regarding the following measures of interest were reviewed from medical charts. Demographic information reviewed included age, sex, body mass index (BMI), history of diabetes mellitus, smoking history, and history of medications (anticholinergics and hypnotics). Functional ambulation ability within the past 3 months was assessed. Before performing VFSS, history taking and physical examination were usually performed and recorded on the chart for patients. Recent functional ambulation ability was identified through the patients’ medical records and nursing records including the VFSS charts. Subjects who cannot ambulate or required a parallel bar or assistance from more than 1 person for ambulation were categorized under nonfunctional ambulation. On the other hand, those who could perform functional ambulation with or without assistance were categorized under functional ambulation.

Diagnosis leading to hospitalization was classified according to the following etiologies: respiratory disease, cancer, cardiac disease, renal disease, sepsis, status post operation, gastrointestinal disease, endocrine disease, and miscellaneous etiologies. We also investigated whether patients were diagnosed with community acquired pneumonia (CAP) upon referral according to the following criteria: suspected symptom of pneumonia acquired before hospitalization, elevated body temperature, abnormal laboratory findings (white blood cell count and inflammation-related protein levels) and abnormal chest radiography findings.

Blood sample results obtained within a week before performing VFSS were reviewed, subsequently selecting the latest values. Serum albumin concentrations, an indicator of nutritional status, were reviewed, with concentrations <3.5 g/dL indicating low serum albumin. The blood urea nitrogen (BUN) to serum creatinine ratio, an indicator of dehydration,^[8]^ was also determined.

History of ICU stay and history of endotracheal intubation during the hospital stay were reviewed upon referral.

### 2.4 Statistical analysis

Statistical analyses were performed using SPSS ver. 19.0 (IBM Corp., Armonk, NY), with *P* < .05 being considered significant. The Mann–Whitney *U* test was used for comparison of demographic information, functional ambulation ability, presence of accompanying CAP, serum albumin concentration, and BUN to serum creatinine ratio, history of ICU stay, history of endotracheal intubation, and VDS scores among groups 1 and 2. Patient diagnoses were reported as frequencies and proportions during descriptive analysis. The effects of independent variables, including functional ambulation ability, serum albumin concentration <3.5 g/dL, and presence of accompanying CAP were evaluated using multivariate logistic regression analysis, with associations being presented as odds ratios (ORs).

## 3 Results

### 3.1 General patient characteristics

Table 1 shows the demographic and clinical characteristics of the 52 patients included herein. Group 1 (patients with airway invasion on VFSS) and Group 2 (patients without airway invasion on VFSS) each had 26 patients. No significant differences in age, sex, BMI, history of diabetes mellitus, smoking history, and history of medications (anticholinergics and hypnotics) were observed between both groups (*P* > .05). Moreover, 11 (42.31%) patients in group 1 and 19 (73.08%) in group 2 were assessed to have functional ambulation, the difference being significant (*P* = .03). Presence of accompanied CAP was significantly greater in group 1 than in group 2 (*P* < .01). Laboratory results showed that group 1 had a lower mean serum albumin concentration than group 2 (*P* = .02). No significant difference in the BUN to serum creatinine ratio (*P* = .07), as well as history of ICU stay and history of endotracheal intubation during the hospital stay (*P* > .05), were observed between both groups (Table 1).

**Table 1:** Demographics and clinical characteristics of non-neurologically ill patients with dysphagia.

	Group 1 (n = 26)	Group 2 (n = 26)	*P* value
Age, yr, mean ± SD	75.27 ± 8.93	73.58 ± 11.59	.56
Sex, male/female, n (%)	18 (69.23)/8 (30.77)	16 (61.54)/10 (38.40)	.56
BMI, mean ± SD	20.2 ± 5.54	21.78 ± 2.81	.24
Diabetes mellitus, n (%)	5 (19.23)	7 (26.92)	.51
Current smoking, n (%)	2 (7.69)	1 (3.85)	.55
Current medication, n (%)			
Anticholinergics	3 (11.54)	3 (11.54)	1.00
Hypnotics	7 (26.92)	2 (7.69)	.07
Functional ambulation Categories, n (%)			.03^∗^
Functional ambulation	7 (26.92)	15 (57.69)	
Nonfunctional ambulation	19 (73.08)	11 (42.31)	
Diagnosis, n (%)			
Respiratory disease	3 (0.12)	3 (0.12)	
Cancer	5 (0.19)	5 (0.19)	
Cardiac disease	2 (0.08)	4 (0.15)	
Renal disease	2 (0.08)	1 (0.04)	
Sepsis	1 (0.04)	2 (0.08)	
Status post operation	3 (0.12)	0 (0.00)	
Gastrointestinal disease	0 (0.00)	1 (0.04)	
Endocrine disease	1 (0.04)	0 (0.00)	
Miscellaneous etiologies	5 (0.19)	5 (0.19)	
CAP, n (%)	16 (61.54)	4 (15.38)	<.01^∗^
Laboratory studies			
Albumin, g/dL, mean ± SD	2.78 ± 0.44	3.38 ± 0.72	<.01^∗^
Albumin, n (%)			<.01^∗^
Albumin ≥3.5g/dL	1 (4.35)	9 (40.91)	
Albumin<3.5g/dL	22 (95.65)	13 (59.09)	
BUN/Cr, mean ± SD	30.45 ± 19.91	21.17 ± 12.27	.07
ICU stay, n (%)	8 (30.77)	4 (15.38)	.19
LOS-ICU, d, mean ± SD	22.75 ± 18.01	13.00 ± 5.72	.33
Intubation, n (%)	7 (26.92)	4 (15.38)	.31

### 3.2 Factors contributing to airway invasion

Table 2 summarizes the ORs between both groups. Multivariate logistic regression analysis revealed that functional ambulation ability was significantly associated with airway invasion (OR: 3.57, 95% CI 1.14–11.19; *P* = .03), suggesting that patients with nonfunctional ambulation had significantly higher risk for airway invasion. Presence of accompanying CAP (OR: 5.06, 95% CI 1.56–16.44; *P* = .01) was significantly associated with airway invasion. Similarly, serum albumin concentration was significantly associated with airway invasion, suggesting that patients with albumin concentration <3.5 g/dL (OR: 4.90, 95% CI 1.39–17.32; *P* = .01) were at higher risk for airway invasion (Table 2).

**Table 2:** Multivariate logistic regression analysis for airway invasion among non-neurologically ill patients with dysphagia.

		OR	95% CI for OR	*P* value
Functional ambulation	No	3.57	1.14, 11.19	.03^∗^
	Yes	1		
CAP	Yes	5.06	1.56, 16.44	.01^∗^
	No	1		
Albumin	<3.5g/dL	4.90	1.39, 17.32	.01^∗^
	≥3.5g/dL	1		

### 3.3 Comparison of associated swallowing process according to VDS

Differences in problems associated with the swallowing process according to VDS scores were compared between both groups with results being presented as mean score **±** standard deviation. Among the oral items, group 1 had significantly higher scores for bolus formation (*P* = .01), tongue-to-palate contact (*P* = .02), and premature bolus loss (*P* < .01) compared to group 2. None of the patients showed apraxia. Among the pharyngeal items, group 1 had significantly higher scores for vallecular residue (*P* = .03) and coating of pharyngeal wall (*P* = .01) compared to group 2 (Table 3).

**Table 3:** Comparison of videofluoroscopic dysphagia scale scored among non-neurologically ill patients with dysphagia.

Item	Group 1 (n = 26)	Group 2 (n = 26)	*P* value
Oral item			
Lip closure	0.23 ± 0.65	0.00 ± 0.00	.08
Bolus formation	1.38 ± 1.53	0.35 ± 1.29	.01^∗^
Mastication	1.23 ± 1.88	0.77 ± 1.97	.39
Apraxia	0.00 ± 0.00	0.00 ± 0.00	
Tongue to palate contact	1.92 ± 2.48	0.38 ± 1.96	.02^∗^
Premature bolus loss	2.42 ± 1.59	1.04 ± 1.18	<.01^∗^
Oral transit time	0.81 ± 1.36	0.58 ± 1.21	.52
Pharyngeal item			
Triggering of pharyngeal swallow	0.69 ± 1.66	0.69 ± 1.66	1.00
Vallecular residue	3.85 ± 1.59	2.85 ± 1.62	.03^∗^
Laryngeal elevation	1.04 ± 2.93	0.35 ± 1.77	.31
Piriformis sinus residue	6.40 ± 3.41	5.02 ± 2.94	.12
Coating of pharyngeal wall	6.58 ± 4.07	3.46 ± 4.47	.01^∗^
Pharyngeal transit time	0.46 ± 1.63	0.69 ± 1.95	.65
Aspiration	10.15 ± 2.82	0.00 ± 0.00	

## 4 Discussion

The current study identified factors associated airway invasion and determined accompanying problems according to VFSS findings among non-neurologically ill patients with dysphagia. Accordingly, our results found that nutritional status and functional ambulation ability were significantly associated with airway invasion, suggesting that patients with airway invasion were significantly more likely to have CAP.

Dysphagia, otherwise known as swallowing difficulty, has been a common clinical problem that increases morbidity and hospital care costs. Although dysphagia can be mainly caused by neuromuscular diseases or structural abnormalities of the oropharynx and esophagus, it has also been observed among patients without such conditions. Studies have reported that dysphagia has a prevalence of 22.6% among non-neurologically ill patients with dysphagia in primary care,^[14]^ 11.4% to 38% among community-dwelling individuals,^[15,16]^ 40% to 60% among nursing home residents,^[17,18]^ and 26.2% among acute-care older patients.^[19]^ Given that airway invasion, 1 of the significant causes of dysphagia, is a critical condition that can cause life-threatening diseases,^[20]^ accurate evaluation and identification of airway invasion, as well as factors associated therewith, is imperative. Recently, while several studies have reported on factors influencing dysphagia symptoms,^[14–19]^ few have utilized imaging evaluation to investigate airway invasion among patients with dysphagia. The current study conducted VFSS to evaluate the presence of airway invasion among non-neurologically ill patients who complained of dysphagia.

VFSS has been the most commonly used method for assessing swallowing dysfunction given that it is the most definitive method for determining the presence and severity of dysphagia by allowing the visualization of the swallowing process.^[21,22]^ VFSS can also detect the presence and timing of airway invasion and helps identify the mechanism through which dysphagia occurs, which can help establish treatment direction.^[23,13]^ We identified clinical characteristics that differed depending on the presence of airway invasion though VFSS findings among patients who complained of dysphagia. Accordingly, our results identified functional ambulation inability, low serum albumin concentration, and presence of accompanied CAP as risk factors for airway invasion among non-neurologically ill patients.

Patients with airway invasion included herein exhibited lower physical function (i.e., functional ambulation inability) compared to those without airway invasion (OR, 3.57; 95% CI, 1.14–11.19; *P* = .03). This result seems to suggest that reduced functional activity affects the risk for airway invasion, which may be associated with sarcopenia. Sarcopenia refers to the degenerative loss of muscle mass and decrease in body strength that can be caused by internal (aging) and external factors (nutritional deficiency and immobilization).^[2]^ Sarcopenia has been reported to be an important risk factor for dysphagia among hospitalized elderly patients.^[24]^ In addition, the results presented herein suggest that sarcopenia further contributes to the development of airway invasion among patients with dysphagia. Moreover, secondary sarcopenia of the entire skeletal muscles, including those related to swallowing process, can considered as a mechanism for the higher risk of airway invasion among individuals with reduced functional activity.

Our results showed that low serum albumin concentration (<3.5 g/dL) (i.e., hypoalbuminemia) was also independently associated with airway invasion (OR, 4.90; 95% CI, 1.39–17.32; *P* = .01) among non-neurologically ill patients with dysphagia. Serum albumin concentration has been one of the most commonly used indicators of nutritional status among older populations.^[25]^ The decreased in food consumption can lead to malnutrition and reduced body weight, subsequently resulting in physical fragility and vulnerability to acute diseases.^[26,27]^ Thus, hypoalbuminemia can also be related to sarcopenia. However, the current study found no significant difference in BMI between the 2 groups. Although BMI is also commonly used as an indicator of nutritional status, it may not accurately reflect sarcopenia. One of the major changes in body composition with aging is an increase in body fat and a decrease in skeletal muscle mass, which may not produce a change in BMI.^[28]^

Additionally, after analyzing the cause of airway invasion among non-neurologically ill patients with dysphagia, we found that the proportion of participants diagnosed with CAP was higher among those with airway invasion group (53.9%) than among those without airway invasion (15.4%). Previous studies have reported that aspiration increases the risk for subsequent pneumonia.^[29]^ Moreover, the results presented herein suggest that accompanying CAP can also be a risk factor for aspiration. The muscles responsible for swallowing are closely connected with the respiratory system. Muscles related to swallowing can be histologically classified as striated muscles but have embryologically different properties from limb skeletal muscles. The muscles that influence the swallowing process include those of the larynx and pharynx, which are commonly formed from the fourth branchial arch. These muscles are controlled by the respiratory center in the brainstem and show synchronized action with exhalation.^[30]^ We suggest that pneumonia interferes with this sequence of processes and may increase the risk for airway invasion.

The results of the current study suggest that the relationship between all 3 factors, including decreased functional ambulation ability, low serum albumin concentration, and presence of accompanying CAP, and airway invasion among non-neurologically ill patients with dysphagia resemble a cogwheel. Accordingly, decreased functional level can lead to eating disorders, which deteriorate nutritional status and lead to malnutrition and reduced albumin concentration. Subsequently, malnutrition leads to more physical fragility and sarcopenia, which affect vulnerability to pneumonia. Sofia et al reported that sarcopenia increased the risk of CAP by 3.88 times.^[31]^ Sarcopenia interferes with the immune system, subsequently inducing immunosuppression by reducing muscle IL-15 formation and causing changes in neutrophil migration and phagocytosis. Furthermore, sarcopenia by itself causes generalized muscle weakness, which affects respiratory muscles. Therefore, monitoring unnecessary bed rest, preventing reduced physical function, and facilitating nutrition management among hospitalized patients is important.

Given the ability of VFSS to evaluate various problems in the oropharyngeal and esophageal phases, as well as penetration and aspiration, it has some advantages in determining which among the swallowing phases is problematic and what type of swallowing therapy should be performed. Recently, the VDS has been used to quantify the severity of swallowing dysfunction in many etiological conditions.^[5,7]^ The current study showed that patients with airway invasion had higher scores associated with bolus formation, tongue-to-palate contact, premature bolus loss, vallecular residue, and coating of pharyngeal wall compared to those without airway invasion. Our results suggested that airway invasion due to dysphagia was associated with an overall reduction in swallowing phases. Previous studies have reported the relationship between whole-body sarcopenia and problems in the oral phase of swallowing.^[3,32]^ Moreover, Machida et al found that sarcopenia affected tongue pressure and jaw opening,^[3]^ while Sakai et al. reported that tongue strength was independently associated with muscle function and nutritional status, both of which may be related to secondary sarcopenia among elderly patients.^[32]^ Additionally, in relation to the pharyngeal phase of swallowing, reduced muscle mass and decreased strength of the swallowing muscles might cause pharyngeal residue resulting from decreased pharyngeal constriction and opening dysfunction of the upper esophageal sphincter.^[2]^

Some limitations of the current study need to be considered. First, though patients aged 18 years and older were included herein, the final groups of subjects analyzed were elderly patients with a mean age 74.4 years old. Thus, our results may not be generalized to all non-neurologically ill patients with dysphagia. Further studies should therefore include patients across a wider age range. Second, the number of cases included in this study was relatively small. Though 52 patients were ultimately included in the analysis, which showed statistical significance, a larger study will be needed to validate these results. Third, given the retrospective nature of the study, there were some difficulties during the collection of comprehensive information from the medical chart. Functional ambulation ability was neither quantified nor further subdivided. Other features of the subjects’ physical examination, history, or imaging findings that affected treatment decisions may not have been recorded in the medical chart and could have caused selection bias.

## 5 Conclusions

In conclusion, the current study identified factors contributing to airway invasion determined through VFSS among non-neurologically ill patients with dysphagia. Although several clinical signs and radiologic findings can help with the diagnosis of dysphagia, airway invasion has been the most important factor. However, few studies have investigated differences in contributing factors and accompanying problems during the swallowing process utilizing VFSS findings among non-neurologically ill patients with dysphagia. Our results showed that airway invasion was 3.6, 4.9, and 5.1 times more likely to occur among non-neurologically ill patients who cannot functionally ambulate, had serum albumin concentrations <3.5 g/dL, and had accompanying CAP, respectively. Our findings suggest improving low activity status and malnutrition as possible methods for preventing airway invasion among older inpatients. Moreover, we recommend that the presence of airway invasion be assessed through VFSS among patients who have lower physical function, low serum albumin concentration, and CAP.

## Author contributions

**Conceptualization:** Hee Kyung Cho.

**Data curation:** Kang Lip Kim, Do Yun Kwon.

**Formal analysis:** Sang Gyu Kwak.

**Investigation:** Kang Lip Kim, Hee Kyung Cho, Do Yun Kwon.

**Supervision:** Gi-Young Park, Dong Rak Kwon.

**Writing – original draft:** Kang Lip Kim, Hee Kyung Cho.

**Writing – review & editing:** Hee Kyung Cho.
